# Intra-individual variation of hen movements is associated with later keel bone fractures in a quasi-commercial aviary

**DOI:** 10.1038/s41598-023-29587-9

**Published:** 2023-02-09

**Authors:** Camille M. Montalcini, Michael J. Toscano, Sabine G. Gebhardt-Henrich, Matthew B. Petelle

**Affiliations:** 1 ZTHZ, Division of Animal Welfare, VPH Institute, University of Bern, 3052 Zollikofen, Switzerland; 2 Graduate School of Cellular and Biomedical Sciences, University of Bern, 3012 Bern, Switzerland

**Keywords:** Ecology, Evolution, Zoology

## Abstract

Measuring intra- and inter-individual variation in movement can bring important insights into the fundamental ecology of animals and their welfare. Although previous studies identified consistent differences in movements of laying hens within commercial aviaries, the level of consistency was not quantified, limiting our capacity to understand the importance of individual movements for welfare. We aimed to quantify the scope of intra- and inter-individual differences in movements of commercial laying hens and examined their associations with indicators of welfare at the end of production. We quantified individual differences in one composite daily movement score for 80 hens over 54 days post-transfer to a quasi-commercial aviary. Results showed consistent inter-individual differences in movement averages, explaining 44% of the variation, as well as individual variation in predictability and temporal plasticity (at the population-level, hens increased their movements for 39 days). Hens that were more predictable in their daily movements had more severe keel bone fractures at the end of production while we found no such correlation between daily movement averages (individual intercept) and welfare indicators. Our findings highlight the importance of inter-individual difference in intra-individual variation of movements to improve poultry welfare.

## Introduction

Intra-individual variation in movements of animals result from a dynamic interplay between factors such as health^1–3^, spatial memory^4,5^, need for resource acquisition^6^, social interactions^7^ and predation risk^8^. The temporal dynamics of these relationships illustrate the complexity of individual variation in movements as well as their importance for the fundamental ecology of animals^9^ and animal welfare. For instance, intra-individual variation in movements could be used as an early warning of health issues in animals^10^ but also to protect endangered species (e.g. when used as a complementary tool by wildlife managers for anti-poaching efforts^8^ and for improving reintroduction success^11^). Quantifying biologically relevant behavioural variation is achieved using methods that decompose phenotypic variation into intra- and inter-individual components. The latter component provides information that is particularly relevant at the group level. For instance, differences between individuals in movements and space use behaviours may facilitate population-level adaptation in animals^12^ as well as access to resources of group-housed animals where not all resources and areas can be accessed by all individuals simultaneously^13–15^.

Until recently, measuring inter-individual variation in movements has been focused on consistent individual differences in averages (personality) or plasticity (i.e. average change in behaviour across a context)^16–18^. Focusing solely on phenotypic averages, however, restricts our understanding of important biological variation. By including residual intra-individual variation in behaviour (predictability), we obtain a more nuanced and comprehensive understanding of individuals^19–21^. To the authors’ knowledge, this component of behavioural variation has never been studied in hen movements and its underlying mechanisms remain generally unclear and understudied^22^. Additionally, we lack quantification of consistent inter-individual differences in both average and variability of hen movements. Previous studies have identified consistent individual differences in daily movement patterns^23^ or space use behaviours^24^ within the interior of aviaries, but repeatability, a population-level measure quantifying the extent of individual differences, was not directly quantified. This gap likely extends from the challenge of monitoring individual movements in a densely populated housing containing multiple stacked horizontal levels (such as aviaries) and the various types of possible movements (e.g. flying, walking) between areas.

Within Europe, battery cages in the poultry industry are being replaced by cage-free systems (e.g. aviaries and free range) that are believed to benefit animal welfare mainly through greater ability to move freely and perform more natural behaviours^25^. Despite benefits, these modern systems, in particular aviaries, have a higher incidence of severe feather pecking, bacterial infections, and keel bone fractures compared to cage-systems^26^. These increased welfare issues in cage-free systems are due to a variety of reasons including the complexity of the structure which increases the risk of collision, and the large group size where bacterial infections or severe feather pecking are harder to detect and control. A better understanding of how individuals acclimate to and behave in this complex environment is a first, but important step to tackle these welfare issues. Because individual movements are indicative of how individuals interact with their environment, tracking systems are a promising tool to identify potential issues in cage-free systems, and improve poultry welfare (e.g. by modifying husbandry practices or housing systems to better allow expression of important behaviours^27^ or reduce prevalence of potentially harmful behaviour^28^).

The transfer from a rearing to a laying barn is a standard practise in poultry farming that may be stressful to the animals. In the following weeks, hens will experience substantial environmental and internal variability (e.g. new husbandry practices, social settings; bone maturation, onset of lay), likely contributing to intra-individual variation in behaviours. Furthermore, these first weeks in the laying barn are important for hens’ welfare and producers, due to an increased mortality risk at the onset of lay^29^. In this study, we quantified the scope of inter-individual differences in averages and in intra-individual variability of a composite score reflecting daily movements within a quasi-commercial aviary, over 54 days post transfer to a laying barn. We evaluated associations between the hen’s welfare assessed at the end of production and both the hen’s average and variability of the movement score. We expected to find individual variation in averages, predictability, and temporal plasticity of the daily movement score. We hypothesized that hens showing less behavioural variability would be behaviourally more constrained and thus less able to behaviourally cope with environmental changes (such as alterations in management practices or social structure). Therefore, we predicted that a high level of predictability or a low level of plasticity in movements would be associated with generally poorer welfare, including more severe keel bone fractures and feather damage.

## Materials and methods

### Experimental design

This study involved initially four rearing pens each containing 630 Dekalb White chicks (*Gallus domesticus*, a widespread hybrid across the world within commercial egg production) from the same parent flock, housed in a standard rearing facility, containing an Inauen Natura aviary described previously^1^. All chicks came from the same commercial hatchery, but, for the purpose of a larger study, half of the chicks (housed in two of the four rearing pens) were hatched on-site within the mentioned rearing barn (on-farm hatch treatment; were transported 3 days before hatching). The other half of the flock underwent standard hatchery processing in the commercial hatchery (standard treatment) with transportation at 1 day of age. We classified all chicks into a more/less explorers’ class (Supplementary Text S1). At 7 days of age, we selected 96 focal chicks (24/rearing pen; random selection of 10 animals amongst the more exploring class, 10 animals amongst less exploring class and four animals amongst the entire population) which we assigned to one of four identical laying pens associated with the rearing treatment (eight laying pens in total). We assigned focal hens to have equal representation of an individual's class and rearing pen throughout the laying phase. Chicks were individually identified with a leg band characterized by a unique color-number combination. At 17 weeks of age (WOA), we transferred all animals to an onsite quasi-commercial laying barn containing a Bolegg Terrace aviary split into 20 identical pens (Supplementary Fig. S1; previously described in Rufener et al.^1^), and an outside covered winter garden (WG) accessible through pop holes (barn schedule detailed in Supplementary Fig. S2). On the day of transfer, we transported focal hens to their pre-selected laying pen with additional non-focal hens randomly selected across the same treatment, for a total of 225 hens/pen. All barns are located at the Aviforum facility in Switzerland where standard animal husbandry practices are used and from which we received pen-level production data. We extracted the average daily number of eggs per live hen (laid inside and outside the nest boxes) and the daily number of dead hens per pen (illustrated in Supplementary Figs. S3–S4). The WG and the litter area under the aviary are closed during the first eight days to encourage hens to use the nest boxes for egg-laying. To control for the external temperature (hereafter temperature) we extracted the temperature from the nearest regional weather station (LSZB, at Belp Airport, Bern, 10.2 km from the barn) via the Wolfram alpha API in python. The study was conducted according to the cantonal and federal regulations for the ethical treatment of experimentally used animals and all procedures were approved by the Bern Cantonal Veterinary Office (BE-45/20).

### Tracking system

To track individuals across different areas within a laying pen, we distinguished five zones with key resources including the three stacked tiers of the aviary (top tier, nest box tier and lower tier), the littered floor and the WG. The top and lower tiers contained feed, water and perches, the nest box tier contained nest boxes and perches, and the litter floor contained litter (illustrated in Supplementary Fig. S1). Due to the animal density, not all hens can be simultaneously in the nest box tier, top tier or WG. We used a low-frequency tracking system with active tags (mass: 28.1 g) enclosed in a backpack mounted on the back of the focal hens to register any movement across zones (transitions). The tracking system is composed of markers that emits low frequency signals (0.125 MHz) through a cable (creating separately enclosed fields for each zone), active tags (mass: 28.1 g) that can receive signals, and readers that communicates through ultra-high frequency (868 MHz) with the tags and a computer. The receiving strength of the signal is then used to determine theoretical distance to each cable, and in turn to each zone (see Montalcini et al.^30^ for detailed description and validation). We collected tracking data from the first day in the laying barn, although subsequent analysis excluded the day of the transfer to keep only fully tracked days (i.e. the first 17 h hours of tracking^1^). We stopped collecting data on day 54 because all focal birds were handled as part of another study which created a stopping point for data collection. We excluded 16 of the 96 individuals from our analysis due to equipment malfunction and death. Due to low battery level or equipment malfunction, some days were excluded for certain hens so that our analysis included 3750 hen-days and 80 hens (average of 47 days/hen with a minimum of 40 days/hen and a maximum of 49 days/hen).

### Movement data

To quantify individual differences in average and variability of movements over time, it is important to use comparable observations throughout. Therefore, we extracted daily movement variables for each hen from when WG access was provided (i.e. from the second week onward). While the artificial light was on, we extracted the percentage of time spent in each of the five zones, the average number of stays in each zone (per hour to account for varying day length), the average travelled vertical distance (total number of the indoor zones crossed, e.g. a hen jumping from the top tier to litter crosses three zones) per hour and whether the hen entered the WG within 15 min after access was provided (scored 0-no/1-yes). While the artificial light was off (i.e., during the hens' night-time) we extracted the sleeping height, measured by the number of stacked tiers underneath the zone where an animal was for most of the night (value from 0 to 3, e.g. hens sleeping: on the top tier get a 3, on the litter get a 0). Because these movement variables may be intrinsically correlated and we aimed to assess how animals differed in their general daily movement without prior assumptions, we reduced these daily variables into one aggregate by extracting a linear composite variable from a correlation-based principal component analysis (PCA) using the psych package^31^ in R. Due to all hens and all weeks not having the same amount of observations as a result of technical issues, we only included the first day of each week in the PCA for each individual (Supplementary Text S2) to ensure the same mass across weeks and individuals while accounting for the variation in movements across time and individuals. Three of the PCA’s principal components had an eigenvalue > 1^32^, and explained respectively 41%, 20% and 14% of the total variation. Among the variable loadings on the first principal component, nine had an absolute value > 0.4 (Supplementary Table S1a). On the first principal component, the percentage of time spent in the top tier loaded strongly in the opposite direction as the travelled vertical distance and the number of stays in both litter and lower perch. The loadings suggested that this first component reflected general movement throughout the indoor area, where a higher score is associated with animals that spent more time on the floor and lower tier but also transitioned more between these zones. We projected all observations onto the subspace spanned by the first principal component to obtain daily movement scores for each hen (PC1). The loadings of the other two principal components (not used in subsequent analysis) suggested two other behavioural axes, one mostly determined by the nest box tier usage and the other axis mostly determined by the WG usage (both in terms of the number of stays and the percentage of time spent; all loadings are detailed in the Supplementary Table S1a).

We then used this daily movement score (PC1) to extract individual-level estimates (intercept, temporal plasticity, and predictability in movements) by gradually increasing the complexity of a linear mixed-effects model and using “best linear unbiased prediction” (BLUPS) to estimate random effects. Furthermore, as we could not extract the daily movement score (PC1) for the first week (due to the WG being closed for management purposes), we extracted an additional individual movement score based on the first week after the transfer to the laying barn, to further investigate the relevance of movements during the early laying phase for animal welfare. Because initial pen-level observations showed a surprisingly high number of birds not transitioning over entire days (34% of hens with no transitions during at least one of the first three days, a value reduced to 1% after 30 days), we extracted individuals’ number of days without transitions between any zones (no-transitions-day). However, the first unintended interruption of the tracking system happened over several days from the 4th day onward, therefore we only used the first 3 days.

### Welfare indicators

Two welfare indicators were assessed on each animal near the end of the production cycle (60 WOA). During the assessment, the two observers were blind to the treatment group (standard versus on-farm hatch), laying pen ID, and hen class. Keel bone fracture severity (KBF) scores (continuous, 0–100) were based on latero-lateral radiographs with a scoring methodology described in Rufener et al.^33^. The KBF score is an indicator of the total amount of bone affected by any fracture in the keel bone. To evaluate inter- and intra-observer reliability, we used the intraclass correlation coefficient with its 95% Confidence Interval (CI). Intra- and inter-observer reliability based on 40 radiographs were high, (ICC = 0.89, 95% CI 0.74–0.95 and ICC = 0.92, 95% CI 0.832–0.96, respectively). A feather damage score (continuous, 0–100) was assigned using the photographs of white laying hens which we rescaled to 0–100 and took the complement to 100 so that higher scores are indicative of poorer welfare (score 1: approx. 100–76 depending on the extent of damage; score 2: approx. 75–51; etc.) for each body part^34^. Lin's concordance correlation coefficient was used to assess intra- and inter-observer reliability (ICC = 0.92, 95% CI 89–94 and ICC = 0.86, 95% CI 0.82–0.89, respectively).

### Statistical analysis

#### Quantifying inter-individual differences in movements

We quantified the extent of inter-individual differences in daily movements (PC1) average response, temporal plasticity, and predictability, by gradually increasing the complexity of a linear mixed-effects model^35^. To quantify inter-individual differences in averages we fitted a random intercept (RI) model to the movement score (PC1) with Hen ID as a random effect to allow the mean response to vary among individuals and to extract individual intercepts. We included as fixed effects: treatment, explorer class, laying pen identity, temperature (°C), time (number of days post-transfer to the barn with starting value 0), and the initial body mass assessed on the day of transfer to the laying barn with a digital scale in grams. Because hens may change their movement behaviour non-linearly across time, we added a quadratic effect of time in the fixed effects. We also accounted for individual differences caused by malfunctioning equipment by adding as a fixed effect hens’ average number of tracked days post-transfer (21–24 days after access to the WG was provided). All continuous variables were scaled and centred to a mean of 0, except for time which was not centred, so that the individual intercept would reflect individual differences in their initial movements (i.e. on the first fully tracked day). To avoid convergence issues in more complex models we excluded the class identity (based on a likelihood-ratio test, LRT) and the laying pen identity random effect (explaining < 1% of the variance not accounted by fixed effects) in subsequent analysis unless otherwise mentioned. To evaluate the magnitude and effect of inter-individual difference in averages, we estimated the adjusted repeatability^36^ by dividing the variance explained by the hen ID by the total phenotypic variance and its 95% credible intervals using 1000 simulations of the posterior distribution of all variance components. For visual purposes, we estimated individual intercept (mean ± sd) with BLUPS by generating 1000 repeated samples from the posterior distributions of the RI model.

To analyse whether individuals also differed in their temporal plasticity, we extended the RI model to a random slope (RS) model, where hen ID is also able to vary across time^37^ (hen ID x time; model named RS1). The inter-individual differences in their temporal plasticity were statistically tested by performing an LRT with the previous RI model. We extended this model including another random slope term allowing individuals to also vary in the curvature of their slope (hen ID × time^2^; model named RS2) and evaluated its significance with an LRT. For RI, RS1 and RS2 models we reported the conditional and marginal R-squared and the Akaike Information Criteria (AIC). We checked model assumptions (i.e., normality of error and homoscedasticity) by sight. For visual purposes, we estimated individual slope (linear, quadratic) (mean ± sd) with BLUPS by generating 1000 repeated samples from the posterior distributions of the RS2 model. We fitted RI, RS and RS2 models with the lme4 package^38^.

To evaluate individual movement differences in predictability we extended the RS2 model to allow estimations of residual intra-individual variation using a double hierarchical model (DHGLM)^39^. We included only significant fixed effects from the RS2 model for both the mean and the dispersion parts of the model. We used a Bayesian Markov Chain Monte Carlo (MCMC) approach using the brms package^40^ in R. We ran the model with uninformative priors, 10 Markov chains, 50,000 MCMC iterations (including 25,000 for burnin), a thinning rate of three and a Gaussian distribution for the response variable (PC1). We extracted the mean and standard deviation from the posterior distribution for each individual’s intercept. Individual estimates were multiplied by − 1 so that individuals with higher estimates have a lower estimated residual variance and are considered as more predictable in their daily movements than individuals with lower estimates. The significance of the variance structure on the dispersion part was statistically tested with an approximate leave-one-out cross-validation (Loo-cv)^41^, comparing the model with one excluding the dispersion part of the model. We quantified the strength of the inter-individual differences in predictability with the coefficient of variation in predictability ($C{V}_{p}$), a standardized population-level measure comparable across studies^35,39^. Specification of the model was assessed with a posterior predictive check, trace plots, the Gelman–Rubin's convergence diagnostic^42^ and Loo-cv.

#### Association with welfare indicators

We fitted two bivariate Bayesian models to evaluate if the identified inter-individual movement differences associated with the KBF severity and feather damage scores, assessed at the end of production. We fitted two bivariate models, one with movement and KBF score as response variable and one with movement and feather damage score as response variables. As fixed effects for movement, we included the time, time^2^, treatment, and temperature. As fixed effects for the welfare indicator, we included the individual predictability estimates and the number of no-transitions-day to estimate their associations with the two welfare indicators, as well as the treatment, the class, and the initial body mass to account for individual differences. We included hen ID as a random effect for both response variables, and individual random slopes (hens ID × time, hens ID × time^2^) for the movement response only. We included laying pen ID as random effect for the welfare response variable. Model parameters were estimated using a Bayesian MCMC sampling method with the MCMCglmm package^43^ for 700,000 iterations with a burn-in phase of 105,000 and a thinning interval of 100. As welfare scores do not have repeated measures at the individual-level we did not allow the variances of the residuals to covary, and we further constrained the residual variance of welfare scores to be fixed and close to zero. Note that to avoid a stats-on-stats issue^44^ when inferring on individuals’ welfare measured on a single instance, with the individual predictability estimate we would need to implement a DHGLM bivariate random regression model with fixed variance structure, which is not supported by the brms package, and so beyond the scope of this paper. We specified priors for the variance structure of the residuals ($R$) with scale equals to a diagonal matrix with entry $1$ for the movement variance and $0.0001$ for the KBF variance, and with degree of belief equal to $1.002$. We specified parameter expanded priors for the variance structures of the random effects ($G1$ for the penID; $G2$ for the HenID) with scale ($V$) equals to a diagonal matrix with $1$ on the diagonal ($I$), degree of belief equal to the dimension of $V$ (i.e. 1 for $G1$, 4 for $G2$) and a multivariate normal prior specification for the redundant working parameters with null mean vector and covariance $I\cdot {25}^{2}$. Further details on the linear model for the latent variable can be found in the Supplementary Eq. S1. Model diagnostics were checked with the trace plots, autocorrelations (< 0.05 for all parameters), and the Gelman and Rubin's convergence diagnostic with 3 chains having over dispersed starting values ($\widehat{R}\le 0.03$ for all parameter)^42^. We deemed a factor or correlation significant if the 95% credible intervals excluded 0.

## Results

### Quantifying inter-individual differences in movements

**Figure 1:**
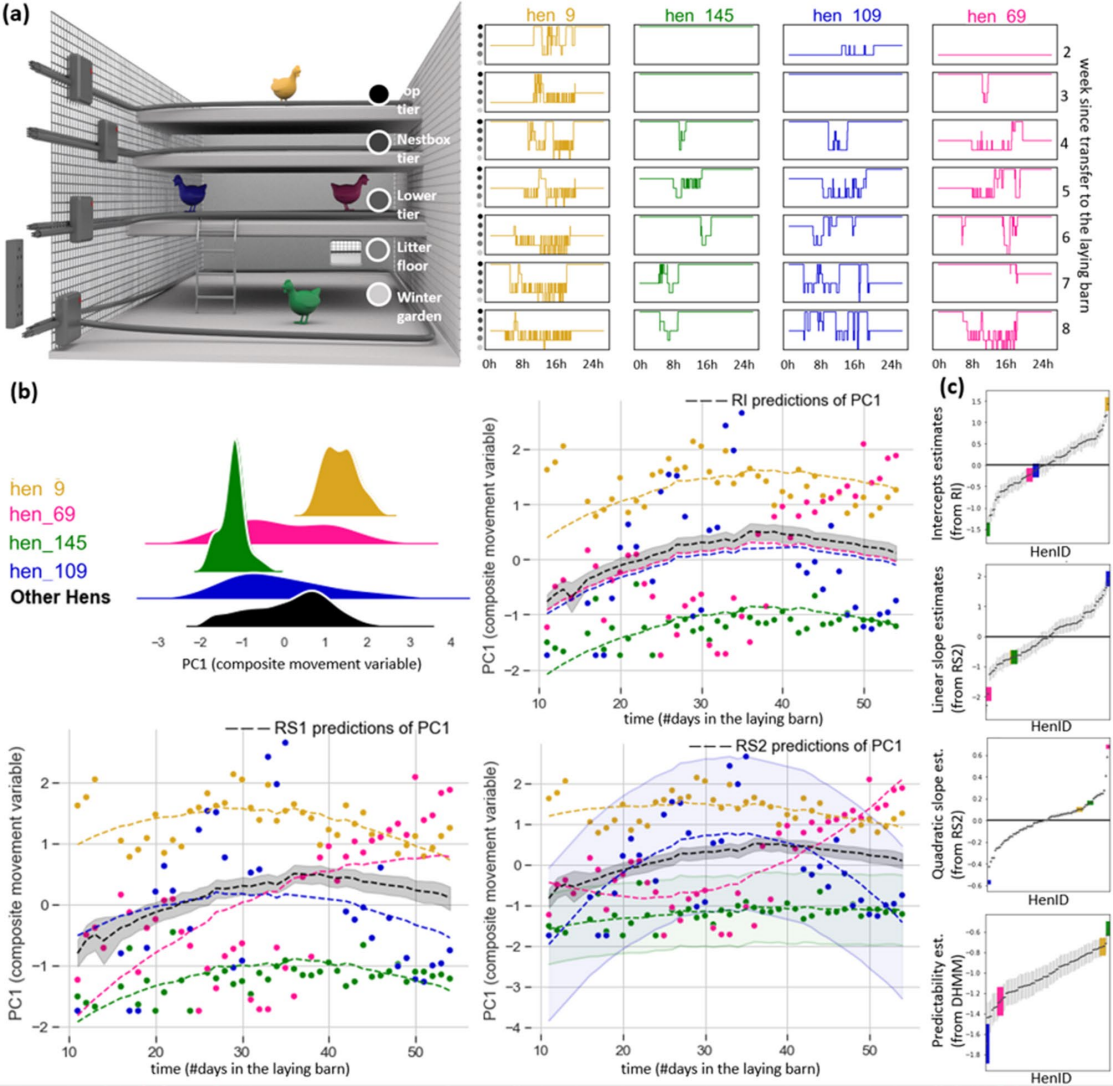
Visual representation of four hens’ transitions across the five aviary zones, daily PC1 scores and individual estimates. Hen 145 and hen 9 have the smallest and largest intercept estimates, respectively. Hen 109 and hen 69 have the smallest and largest quadratic slope estimates, respectively. (**a**) On the left side we showed a simplified representation of the laying barn and the tracking system equipment (dark grey) and on the right side we showed seven (one per week) daily time series of the hens’ transitions across the five aviary zones. Each column represents one hen and each row represents the first tracked day of a particular week. (**b**) PC1 daily scores of four hens including a kernel density estimate (top left); the daily PC1 score over time (dots); the PC1 predictions from studied models (dashed lines); shaded areas around the prediction of two hens to illustrate predictability estimates (represented by a constant value = $\underset{i\,\in\,day}{\mathrm{max}}\left(\left|{prediction}_{i}-{observed}_{i}\right|\right)$). (**c**) Individual estimates (mean ± sd) of studied hens (intercept, linear slope, quadratic slope, and predictability) sorted by the smallest to the highest estimates.

Hens differed in their average movement (repeatability = 0.44, 95% CI 0.43–0.48), meaning that, on average, 44% of the remaining variance (after controlling for fixed effects) in movement can be attributed to differences between individuals. Hens also exhibited differences in temporal plasticity (both linearly and quadratically). Random effects variance estimates for both RI, RS, and RS2 are further detailed in Supplementary Table S2. In addition to inter-individual differences in average and temporal plasticity, results showed that hens differed in their predictability, exhibiting differences in their within-individual variability of behaviour around the mean, with a coefficient of variation in predictability among individuals ($C{V}_{p}$ = 0.25, 95% CI 0.20–0.30). Modelling the dispersion part in addition to the mean part of movement improved the model. We found a negative correlation (${r}_{model name}$) between individual intercepts and linear random slopes (${r}_{RS1}$ = − 0.79, bootstrap 95% CI − 0.82 to − 0.78; ${r}_{RS2}$ = − 0.69, bootstrap 95% CI − 0.76 to − 0.66; ${r}_{\mathrm{DHMM}}$ = − 0.69, 95% CI − 0.80 to − 0.55), a positive correlation between random intercept and quadratic random slope (${r}_{RS2}=$ 0.41, bootstrap 95% CI 0.37–0.54; ${r}_{\mathrm{DHMM}}$ = 0.42, 95% CI 0.21–0.60) and a negative correlation between linear and quadratic random slopes (${r}_{RS2}$ = − 0.89, bootstrap 95% CI − 0.93 to 0.89; ${r}_{\mathrm{DHMM}}$= − 0.90, 95% CI − 0.94 to − 0.84). These results suggest that hens with initially lower movement increased their movement more rapidly than hens with higher initial movement. We found no correlation between predictability and the other individual-level metrics (intercept: ${r}_{\mathrm{DHMM}}$ = − 0.02, 95% CI − 0.26 to 0.23, linear slope: ${r}_{\mathrm{DHMM}}$ = 0.10, 95% CI − 0.16 to 0.34 and quadratic slope: ${r}_{\mathrm{DHMM}}$ = − 0.08, 95% CI − 0.33 to 0.17). Figure 1 illustrates these results by highlighting four daily time series of hens’ initial movements (a), daily PC1 scores with the RI, RS1 and RS2 models predictions, and (b) individual intercepts, slopes and predictability estimates relatively to all other hens (c).

In addition to individual-level variation, we found both linear and quadratic effects of time at the population level. The population, overall, increased their movement until day 39 in the laying barn, at which point, their activity started to decrease (illustrated by the black line in Fig. 1b; Supplementary Table 3). We also found individuals hatched on-farm moved on average less than individuals that were transported at one day of age to the rearing barn, and hens moved generally less within the aviary as the temperature outside increased. Results from fixed effects from RI, RS1 and RS2 models are detailed in Supplementary Table S3.

### Association with welfare indicators

Descriptive statistics of the KBF severity and the feather damage scores are given in Table 1.

**Table 1:** Descriptive statistics of the KBF severity and the feather damage scores assessed at 60 WOA on the 80 hens used in the models.

Welfare indicator	Mean	Standard deviation	Min	0.25 percentile	Median	0.75 percentile	Max
KBF severity	36	15	6	24	36	45	84
Feather damage	34	11	13	27	34	42	65

**Figure 2:**
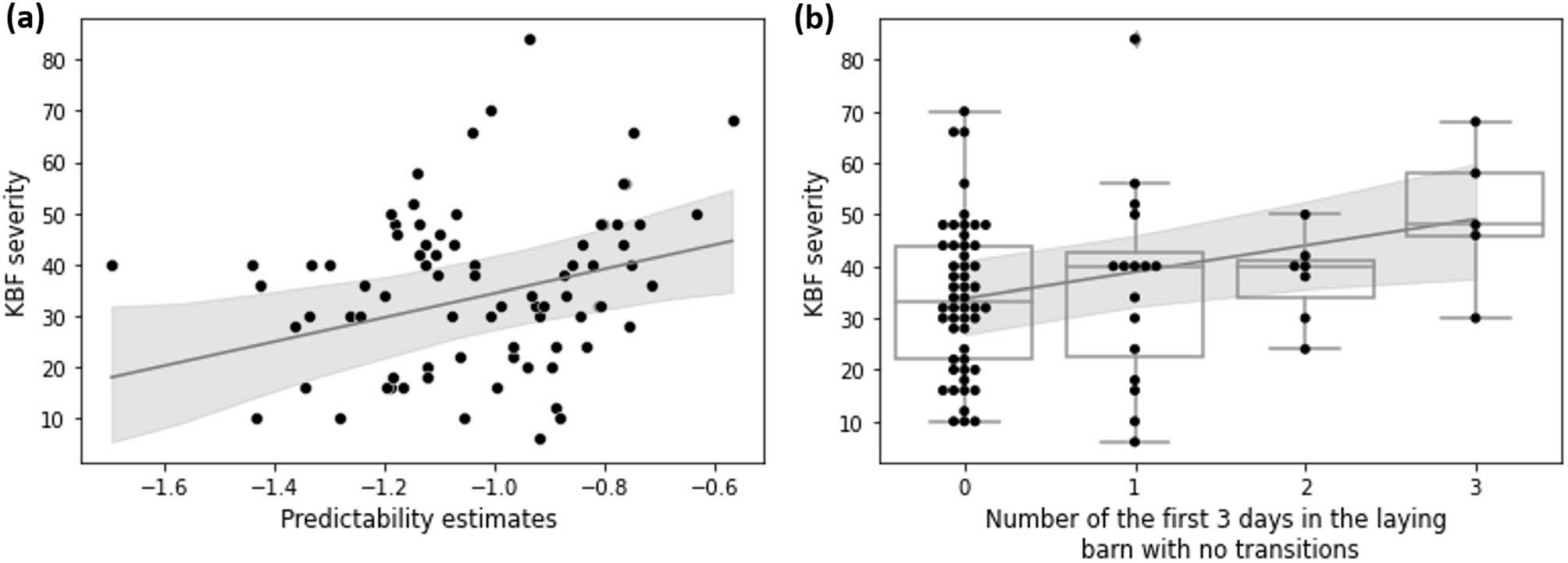
Raw data points of KBF severity in relation to individual predictability (**a**) and the number of days with no transitions during the first three days in the laying barn (**b**), together with the predictions line and its 95% credible intervals from the bivariate model.

Individuals’ predictability of movements had a positive association with individuals’ KBF severity (posterior mean = 23.54, 95% CI 6.57–38.70, Supplementary Table S4), individuals that were more predictable had greater KBF scores (Fig. 2). We did not find correlations between KBF severity and individual intercepts ($r$ = 0.06, 95% CI = − 0.22 to 0.26) or individual linear slopes ($r$ = 0.18, 95% CI − 0.05 to 0.44), but did find a weak negative correlation between KBF severity and individual quadratic slope ($r$ = − 0.24, 95% CI − 0.51 to − 0.04). Furthermore, hens that had a higher number of no-transitions-day during the first three days had higher KBF severity at the end of production (posterior mean = 5.12, 95% CI 1.24–8.69, Supplementary Table S4, Fig. 2).

We did not find an association between individuals’ feather damage and individuals’ predictability nor the number of no-transitions-day during the first week. We also found no correlations between feather damage score and individual intercepts, linear, and quadratic slopes ($r$ = − 0.01, 95% CI − 0.27 to 0.24, $r$ = − 0.22, 95% CI − 0.42 to 0.11, and $r$ = − 0.26, 95% CI − 0.06 to 0.45, respectively). Estimates from fixed and random effects are detailed in Supplementary Table S4 and Supplementary Eqs. S2–S3 from the supplementary materials.

## Discussion

Consistent individual differences in movement behaviour in both wild and domestic species have only recently received attention^9,35,45,46^. Previous studies have identified consistent differences in daily movement patterns^23^ or space use behaviours^24^ of laying hens within aviaries. In the current study, we extended that understanding by quantifying the extent of inter-individual differences in averages (with repeatability) and variability (with the coefficient of variation in predictability) of daily movements using two population-level measures that are, to some extent, comparable across traits and studies. We believe the current effort is the first to reveal intra-individual variability of laying hen movements across time and around their behavioural mean within an aviary. We also found associations between these individual movement scores, assessed during the first two months in an aviary, and the severity of keel bone fractures at the end of production. Collectively, our results highlight the importance of movements during the early laying phase and of the intra-individual variation in movements to explain occurrence of keel bone fractures at end of lay.

We found consistent inter-individual differences of hen daily movements within the first two months in a laying barn, with 44% of the variation attributed to individual differences (repeatability (R) = 0.44). Our study's repeatability is slightly higher than the average reported repeatability of behaviours (across eight taxa: R = 0.37)^47^ and lower than the average repeatability based solely on spatial behaviours (across five taxa: R = 0.67)^12^. Various covariates may explain difference in repeatabilities, including individuals’ life stage^12^. For instance, a meta-analysis conducted by Stuber et al.^12^ found evidence that repeatability increased with increased age, so that difference between individuals during adulthood explained on average about 30% more of the behaviours’ variance compared to juveniles. In this study, we used a transitional life stage between juvenile and adulthood, where individuals are probably still developing cognitively and physiologically as well as gaining spatial experience in their new housing and thus adjusting their behaviours. Therefore, individuals in this study likely exhibited a higher within-individual variation and thus lower repeatability compared to later stages.

After controlling for individual differences in average and temporal plasticity, hens still differed in how predictable they were in daily movements with a relatively low degree of variation within our population ($C{V}_{p}=$ 0.25) but similar to previous results on movement distance (total distance travelled of calves *Bos taurus*: $C{V}_{p}=0.18$^48^ and mean daily distance of wild African elephants *Loxodonta africanus*: $C{V}_{p}=$ 0.27^35^). Quantifying the degree to which individuals vary in their movements within commercial aviaries may be particularly important in light of the restricted space and the high density of animals. Further research is required to understand if a high degree of variation in specific spatial behaviours among individuals housed together would facilitate access to resources and affects animals’ welfare and productivity (such as the timing of nest box usage if it reflects mean oviposition time where maintaining variability could limit competition for nest boxes^15^).

In addition to the two population-level measures (R and $C{V}_{p}$) estimating the degree of individual variation, we found both linear and quadratic effects of time at the population level. Overall, individuals increased their movement until day 39 in the laying barn, that is five days before the last change in the light schedule, near the end of the mortality peak (on average 2.6% of hens died in the studied pens during the first 54 days in the laying barn) and around the moment hens reached maximum production level. It is likely that the egg laying behaviour was an important factor driving the temporal plasticity in daily movements (encouraging them to move to the nest box tier) as well as stress (suggested by a mortality peak and by the high number of hens not transitioning over entire days from the top tier). We also identified differences between individuals in the rate at which they adjusted movement in the laying barn (i.e. temporal plasticity), suggesting that the effect of time at the population level will not capture all relevant information for each individual. The negative correlation between the random intercept and linear slope indicates that individuals with fewer initial movements had a more rapid increase in movement over time as compared to individuals with initially more movements, which may be explained by a regression towards the mean.

The absence of correlations between individuals’ predictability and the other individual-level effects (intercept, linear or quadratic slopes) highlights the relevance of a multidimensional approach to study inter-individual differences in movement that would account for intra-individual variability in addition to individual average response. We also found that individuals exhibiting higher predictability in daily movements had more severe KBF at 60 WOA, supporting our hypothesis that hens with less variability in daily movements may be more constrained in their ability to behaviourally adapt to the commercial environment and in turn have a reduced welfare, compared to less predictable animals. A previous study suggested that space use in the laying barn may be related to differences in spatial cognitive abilities, with hens that never went outside showing lower spatial abilities^5^. Animals with less predictable daily movements may need greater abilities to maneuver in changing conditions (social and environmental) and thus would have greater spatial cognitive ability. Greater spatial cognitive ability would in turn allow individuals to navigate better, leading to lower collision rates with internal structures, a potential cause of KBF^28,49^.

We found no association between temporal plasticity (linear or quadratic) or predictability in daily movements and feather damage. These results suggest that greater variability in movements and possibly more behavioural flexibility to adapt to new social constraints, did not allow hens to better avoid feather pecking, perform maintenance behaviours, or more generally, avoid feather damage. Alternatively, the absence of an association could be explained by the little variation in feather damage between individuals (i.e. most animals had low feather damage). The few studies that have reported associations between individual movements and feather damage (so that hens with greater feather damage used the outside area less frequently^3^) where based on free-range housings which have important differences with our study that contained a WG but no free-range area (i.e., no access to grass and uncovered areas). Also, the WG usage is not well represented by our composite movement score (PC1). More studies are needed to confirm that an animal’s predictability in its daily movement routine associates positively with KBF, but not with feather damage, and whether spatial cognitive ability could explain the association.

Individuals with greater number of no-transitions-day had more severe KBF at the end of production. Every day a hen did not transition between tiers, the hen remained on the top tier, a zone with all essential resources, many perching locations but that lacks important resources (litter, nest boxes and direct UV sunlight). Although reduced accessibility to all resources may compromise an animal's welfare, it is unlikely that this would explain the increased KBF observed at the end of production. Baur et al.^50^ observed almost no KBF at 22 WOA in hens housed in the same barn, which suggest that these days spent on the top tier were not a consequence of KBF but rather expressed in response to the new environment. Because Rufener et al.^1^ found an increased duration of stay in the top tier with increasing KBF severity, it is possible that the top tier is used to offset stress in response to aversive situations such as the transfer to a barn or severe KBF (e.g. by using the perches to avoid more dominant bird^51^). Other locations, such as the nest box, are known to be used in daytime by hens to hide and escape aggression^51^, and the top tier may offer similar refuge as it is accessible at any time (while nest boxes are typically closed in the afternoon).

Altogether, our findings support the relevance of hen movements during the early laying phase, in terms of predictability in daily movements and the number of days spent without transitioning between any aviary’s tiers, to explain later KBF (both movement scores associated positively with KBF score), but further research is required to disentangle underlying mechanisms. Previous literature suggested that the prevalence of KBF increases with the spatial complexity and height of the housing system as well as the presence of perches^52^, and reported KBF to be associated with decreased mobility^53,54^. Although, hens that did not transition for an entire day remained on the top tier (the highest tier with many perching locations) we believe it is unlikely that these movements affect the keel bone directly since there is a gap of 250 days between data collections.

Instead, we propose that these movement scores are expressed as part of a proactive/reactive coping style^55^ that remain consistent through an animal’s life, and which would be associated with both hens’ behaviours and welfare. From a neuroendocrinological perspective, more proactive animals may have less inhibitory control and in turn may be more predictable behaviourally compared to reactive animals^56^, which would be more flexible and perform better under unpredictable environmental conditions^57^. Therefore, hens with less predictable movements may have a reactive coping style and as such be more able to adjust their behaviour to the new environment compared to hens with more predictable movements, which could explain these long-term association to KBF. Further research is required to understand how coping style relate to farm animal welfare, such as KBF, though our methods offer a novel approach to aid welfare assessments.

Exploring coping behaviour in farm animals can provide valuable information to improve animal welfare^55,58^ by optimising husbandry practices and allow individuals to perform effective coping behaviour^58^ in the laying barn. Our study is in line with previous research showing an increased mortality risk at the onset of lay^29^, and further suggest that during the early laying phase some areas may be overcrowded (here, the top tier) or not fully utilized. These findings provide new information that may help to better design and properly prepare hens for inhabiting three-dimensional systems in the future. Furthermore, exploring proxies of coping style (such as potentially predictability in movements) may in the future help to tentatively breed for more resilient farm animals^55,58^. However, the heritability of plasticity and predictability of behaviour or stress responsiveness is still relatively unstudied^59,60^ and will need to be investigated to determine the relative benefits.

## Conclusion

The present study revealed the presence of consistent inter-individual differences in average movements of hens as well as individual variation in predictability and temporal plasticity within the first two months in a quasi-commercial aviary. We found associations of intra-individual variability in daily movement with the severity of KBF and observed a mortality peak and a high number of hens not transitioning over entire days early on. Altogether, these findings highlight the importance of the early laying phase for animal welfare and revealed considerable individual differences providing new information that may help to better design and properly prepare hens for inhabiting three-dimensional systems in the future.

## Supplementary Information

Supplementary Information.

## Data Availability

The data and code for this study are available on https://doi.org/10.17605/OSF.IO/6ARNT.
