# Supplementary material for: Intra-individual variation of hen movements is associated with later keel bone fractures in a quasi-commercial aviary
Source: Sci Rep. 2023 Feb 9;13:2377. doi: 10.1038/s41598-023-29587-9 (PMC9911743; doi:10.1038/s41598-023-29587-9)
Supplement: Supplementary file 1 — Supplementary Information. [file 41598_2023_29587_MOESM1_ESM.docx]

Intra-individual variation of hen movements is associated with later keel bone fractures in a quasi-commercial aviary

Camille Montalcini^1,2^, Michael J. Toscano^1^, Sabine G. Gebhardt-Henrich^1^ and Matthew Petelle^1^

1. ZTHZ, Division of Animal Welfare, VPH Institute, University of Bern, 3052 Zollikofen, Switzerland
2. Graduate School of Cellular and Biomedical Sciences, University of Bern, 3012 Bern, Switzerland

**Supplementary Text S1**

**Chick exploration Class:**

Focal animals were part of a larger study, which classified chicks as more/less explorative (MEXP/LEXP). When chicks were one day of age, a raised platform with blue natural dye was added in each pen, so that chicks going onto the raised platform would have their feet painted in blue. We added blue dye diluted in water approximately every two hours to prevent the dye from drying out. We selected focal animals based on their feet color (blue color: MEXP, no blue color: LEXP) at 7 days of age (this corresponded to when at least 30 animals from each pen had walked onto the raised platform. In each pen, 20 focal animals were selected randomly so that half would belong to the MEXP class and half to the LEXP class (depending on the number of chicks from each class available, the number of animals per class could vary between 6-14). In addition, 20 focal animals were selected as a representative sample of the population (hereafter called OTHERS) (balanced randomization approach aiming at a same proportion of MEXP as in the overall population of that pen). In parallel to the selection process, each selected chick was attributed to a laying pen identity with a balanced randomization approach aiming at a uniform representation in each laying pen of the chicks’ categories (MEXP, LEXP, OTHERS) and rearing pen identity.

**Supplementary Text S2**

**Principal component analysis:**

Because not all hens and all weeks have the same amount of observation, to ensure a same weight across weeks and individuals while accounting for the variation in movements across time and individuals, we included in the PCA each first observation of a week only, for each week and each individual. The first three PCA’s principal components had an eigenvalue > 1 ^1^ and explained 41%, 20% and resp. 14% of the total variation. We calculated loadings for each variable on a given principal component by using the correlations between each original variable and the components ^2^. Among the variable loadings on the first principal component nine had absolute value > 0.4, and six had absolute value > 0.7. Principal component one was characterized by the percentage of duration in the top tier, which loaded strongly in opposite direction as the hourly realized travel distance and as the number of stay in both litter and lower perch. These loadings reflect general movement throughout the barn, with higher score associated with animals that spent more time in the floor, lower tier but also transitioned more between these zones. In contrast, PC2 had the greatest positive loadings on the number of stay in the nest box tier, which could reflect egg-laying behaviour, while PC3 was associated solely with the three WG variables (with loadings > 0.6, all other variables <0.4). This further enhances the meaning and validity of the PCA results^3^. We further evaluated the validity of the PCA by comparing the loadings of the first three principal components (detailed in Table S1 a) with the principal components generated by a PCA based on the full observations set (detailed in Table S1 b).

**Supplementary Equation S1**

**Bivariate mixed models:**

The linear model for the latent variables of the Bayesian bivariate model is defined as: $X\beta+Zu+e$, with $X$ and $Z$ two design matrices relating the fixed and random predictions, respectively, to the data, while $\beta$, $u$ and $e$ (residual) are parameters to estimate. Latter parameters are assumed to follow a multivariate normal distribution:

$\left( \begin{aligned} \beta\\ u \\ ⅇ \end{aligned} \right)\sim N\left( \left[ \begin{aligned} \beta_{0} \\ 0 \\ 0 \end{aligned} \right],\left[ \begin{matrix} B & 0 & 0 \\ 0 & G & 0 \\ 0 & 0 & R \end{matrix} \right] \right)$ (S1)

where the zero off-diagonal matrices imply *a priori* independence between fixed effects, random effects as well as residuals; $\beta_{0}$ and $B$ are the fixed effects’ prior means and respectively (co)variances; $G$ the expected variances ($V$) and covariances ($Cov$) of the random effects:

$G_{HenID}=\left( \begin{matrix} V_{KBF Intercept(Int)} & {Cov}_{KBF Int, PC1 Int} & {Cov}_{KBF Int, PC1:time} & {Cov}_{KBF Int, PC1:time^{2}} \\ * & V_{PC1 Int} & {Cov}_{PC1 Int, PC1:time} & {Cov}_{PC1 Int, PC1:time^{2}} \\ * & * & V_{PC1:time} & {Cov}_{PC1:time, PC1:time^{2}} \\ * & * & * & V_{PC1:time^{2}} \end{matrix} \right)$,

$$G_{PenID}=V_{KBF Intercept(Int)}$$

and $R$ denote the expected variances of the residuals (as health scores do not have repeated measures at the individual-level we do not allow the variances to covary, and we further constrain the residual variance of health scores to be very close to zero):

$R=\left( \begin{matrix} V_{PC1 residual} & 0 \\ 0 & V_{KBF residual} \end{matrix} \right)$.

**Supplementary Equation S2 – S3**

**Bivariate random effects:**

Random effects and residual output from the Bivariate (movement, KBF severity) model:

$\left( \begin{aligned} \beta\\ u \\ ⅇ \end{aligned} \right)\sim N\left( \left[ \begin{aligned} \beta_{0} \\ 0 \\ 0 \end{aligned} \right],\left[ \begin{matrix} B & 0 & 0 \\ 0 & G & 0 \\ 0 & 0 & R \end{matrix} \right] \right)$ (S2)

$$G_{HenID}=\left( \begin{matrix} 213.518 [150.301, 285.914] & 0.361 [-3.818, 4.377] & 2.491 [-0.908,5.917] & -0.867 [-1.734, -0.030] \\ * & 1.231 [0.842, 1.640] & -0.659 [-0.961, -0.381] & 0.092 [0.031, 0.158] \\ * & * & 0.794 [0.532, 1.091] & -0.171 [-0.240, -0.030] \\ * & * & * & 0.047 [0.031,0.064] \end{matrix} \right)$$

$$G_{PenID}=2.531 [0.038, 10.16]$$

$$R=\left( \begin{matrix} 0.259 [0.247, 0.273] & 0 \\ 0 & 0.001 [0.001, 0.001] \end{matrix} \right)$$

Random effects and residual output from the Bivariate (movement, feather damage) model:

$\left( \begin{aligned} \beta\\ u \\ ⅇ \end{aligned} \right)\sim N\left( \left[ \begin{aligned} \beta_{0} \\ 0 \\ 0 \end{aligned} \right],\left[ \begin{matrix} B & 0 & 0 \\ 0 & G & 0 \\ 0 & 0 & R \end{matrix} \right] \right)$ (S3)

$$G_{HenID}=\left( \begin{matrix} 103.808 [69.970, 143.350] & -0.114 [-3.152, 2.849] & -1.423 [-3.946, 1.154] & 0.453 [-0.163, 1.069] \\ * & 1.231 [0.852, 1.653] & -0.660 [-0.965, -0.389] & 0.093 [0.030, 0.162] \\ * & * & 0.797 [0.540, 1.105] & -0.172 [-0.242, -0.112] \\ * & * & * & 0.047 [0.031, 0.065] \end{matrix} \right)$$

$$G_{PenID}=30.59 [0.105,86.08]$$

$$R=\left( \begin{matrix} 0.259 [0.250, 0.272] & 0 \\ 0 & 0.001 [0.001, 0.001] \end{matrix} \right)$$

**Supplementary Table S1**

(a) (b)


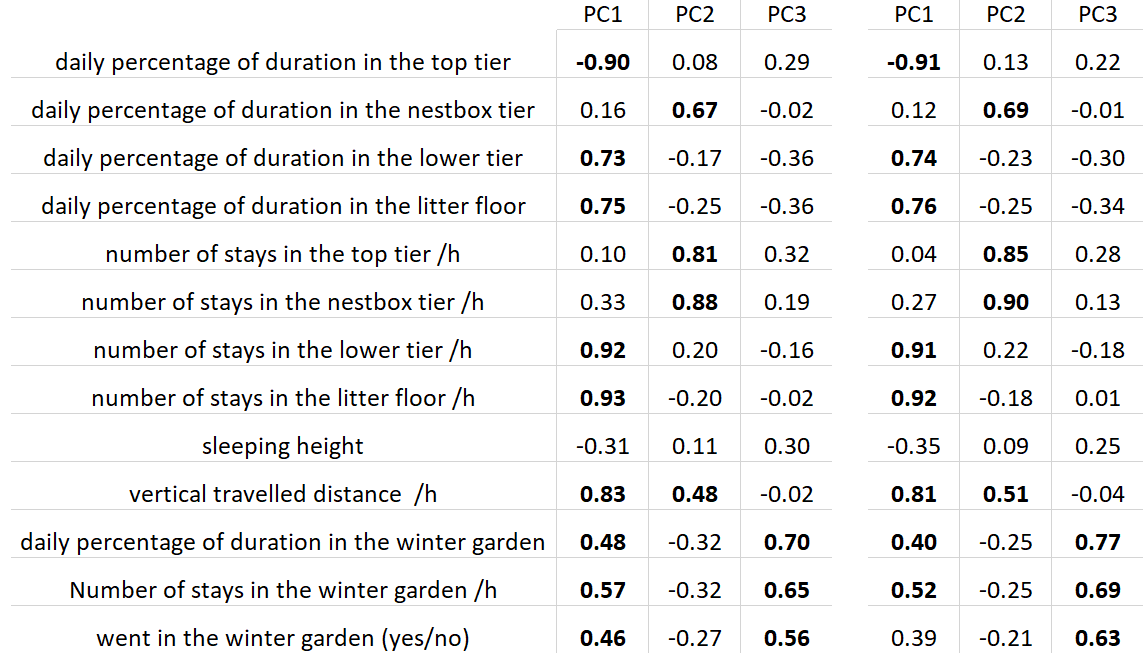


Table S2 – Summary of the principal component analysis (PCA) based on: (a) one value per week per hen, (b) the full set of observations for validation purposes. Loadings with an absolute value over 0.4 are highlighted in bold.

**Supplementary Table S2**


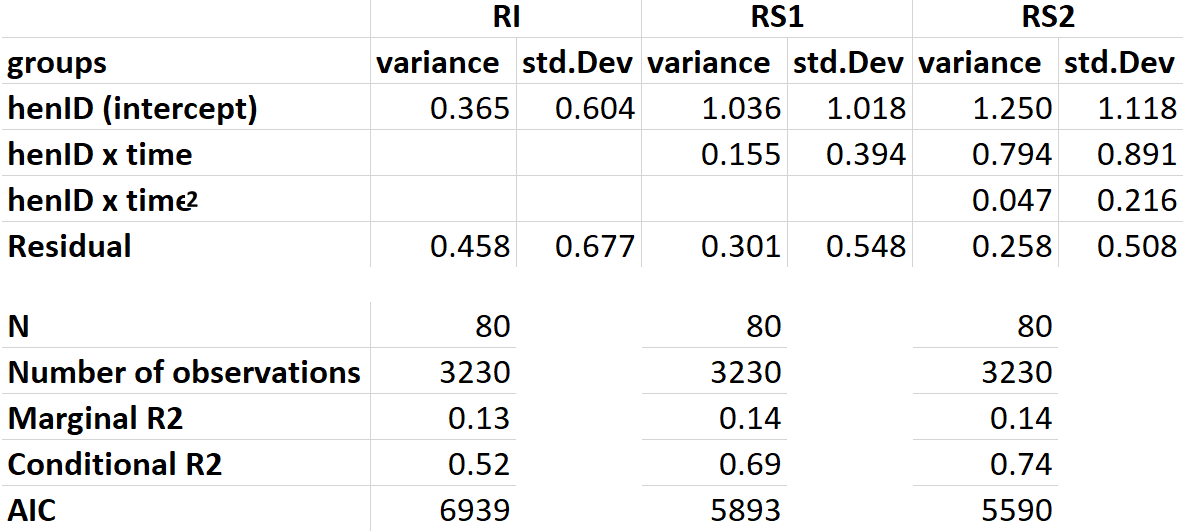


Table S2 – Variance estimates with their standard deviation, R^2^ and AIC values for the random intercept (RI) and the two random slope models (RS1 and RS2). RS1 is a random slope model where time is linear (hen ID x time), and RS2 includes an additional quadratic term of time (hen ID x time^2^).

**Supplementary Table S3**


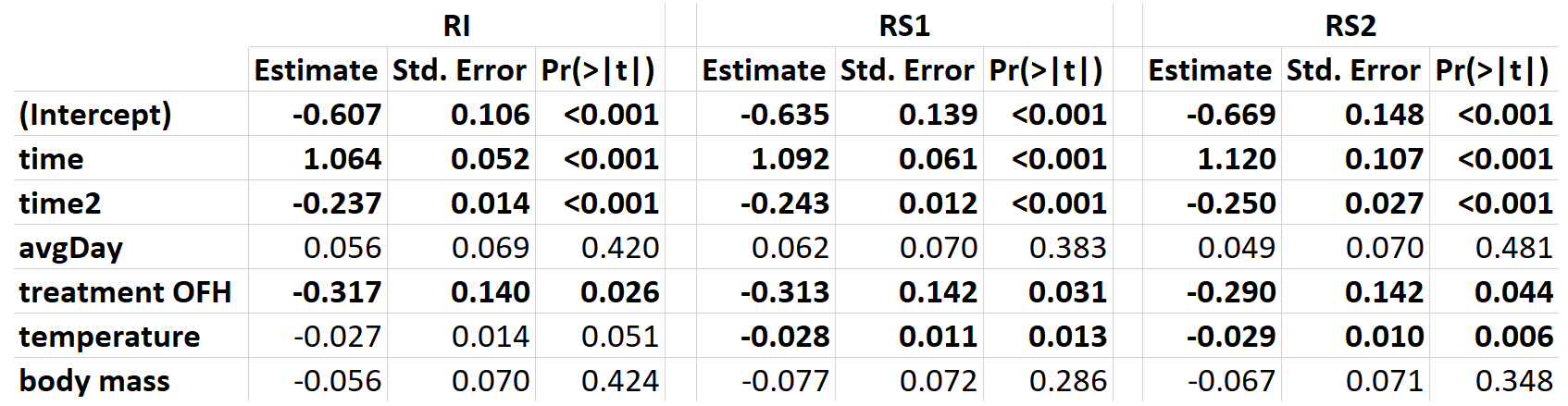


Table S3 – Estimates, standard errors and p-values for random intercept (RI) and random slope models (RS1 and RS2). RS1 is a random slope model where time is linear (hen ID x time), and RS2 includes an additional quadratic term of time (hen ID x time^2^). Significant effects are highlighted in bold.

**Supplementary Table S4**


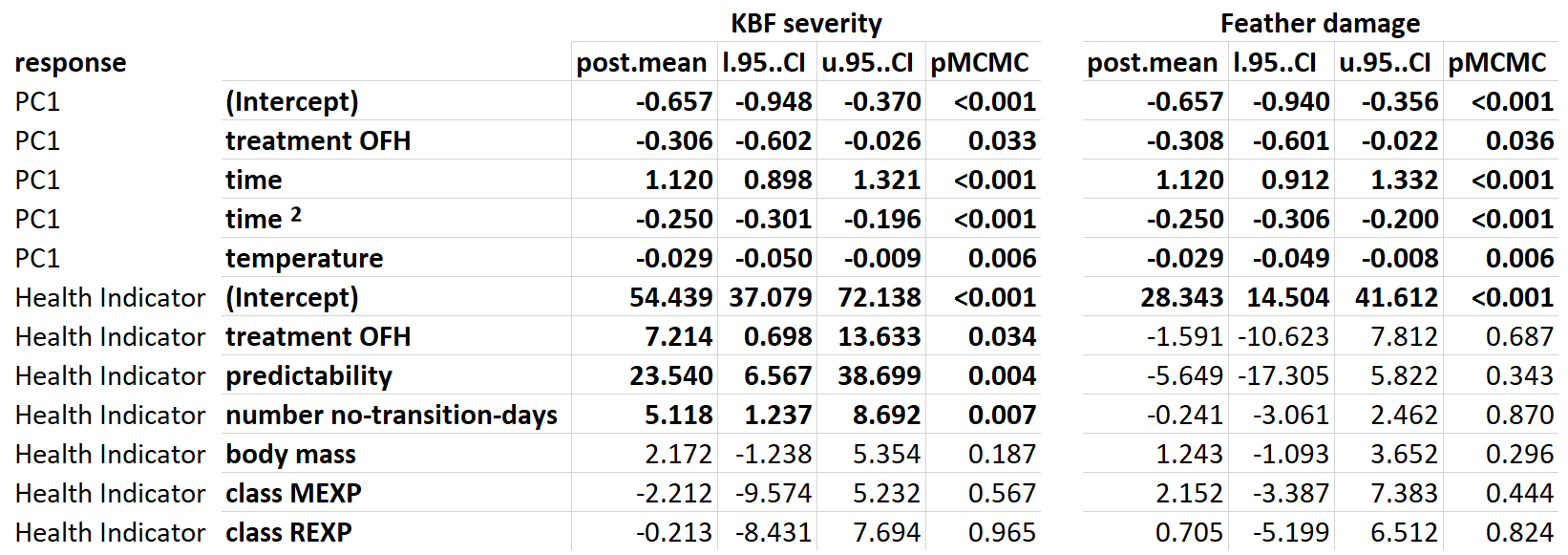


Table S4 – Estimates, 95% credible intervals, and pMCMC for both KBF/PC1 and feather damage/PC1 bivariate models. Significant effects are highlighted in bold.

**Supplementary Figure S1**


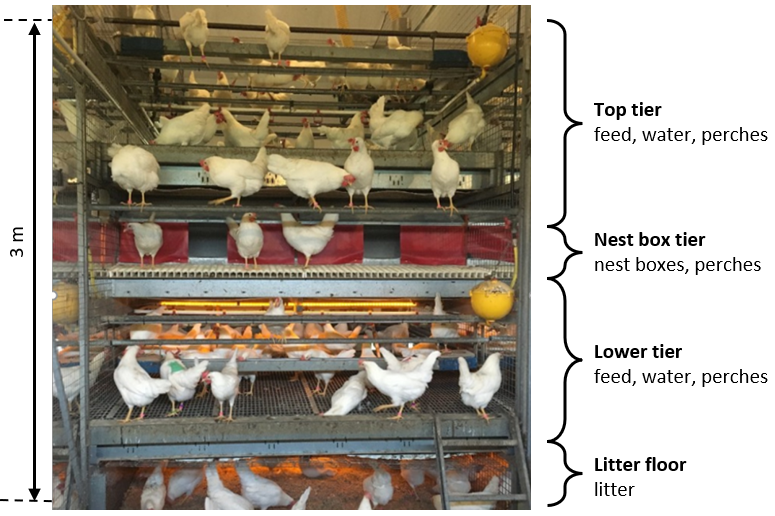


Figure S1 –Side view of the aviary within one pen with its three aviary tiers (top tier, nest box tier and lower tier) and the littered floor


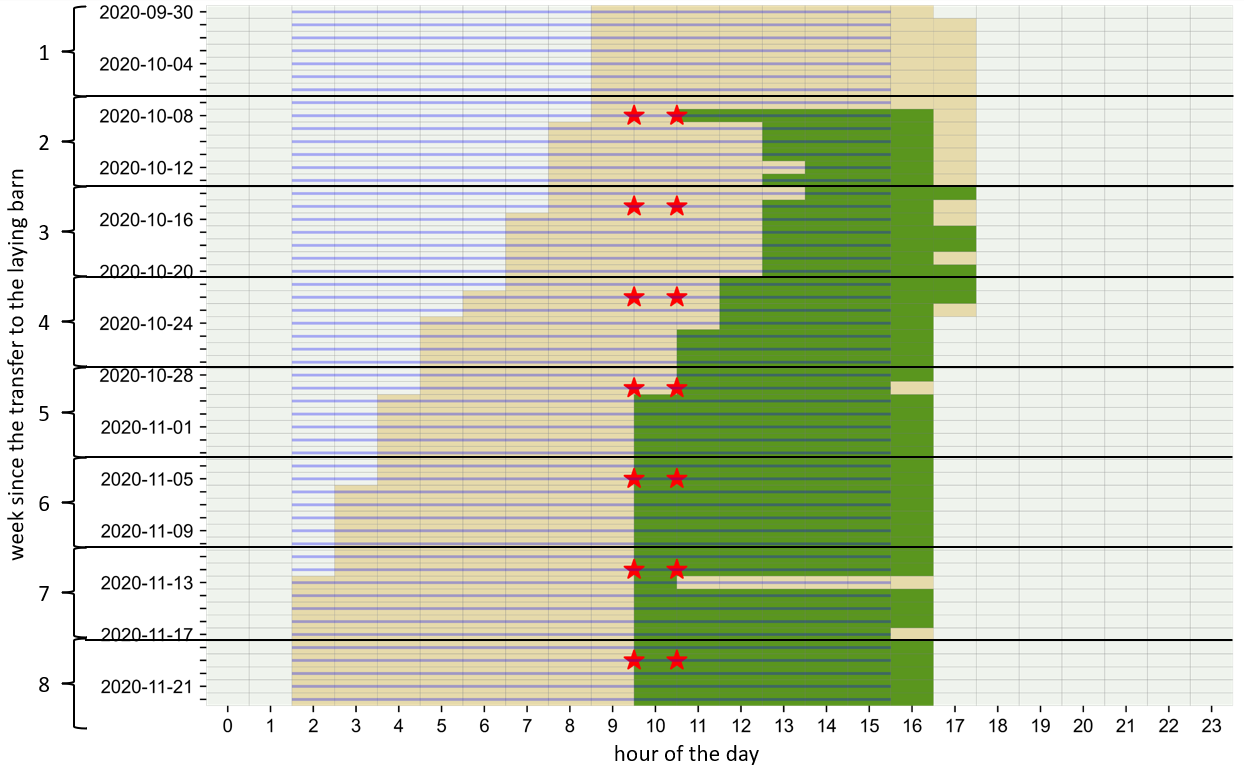
Figure S2 – Daily barn schedule where beige rectangles indicate period where the artificial light was on, red stars indicate when the manure belt was activated, and the green rectangles indicate period where the winter garden was accessible.


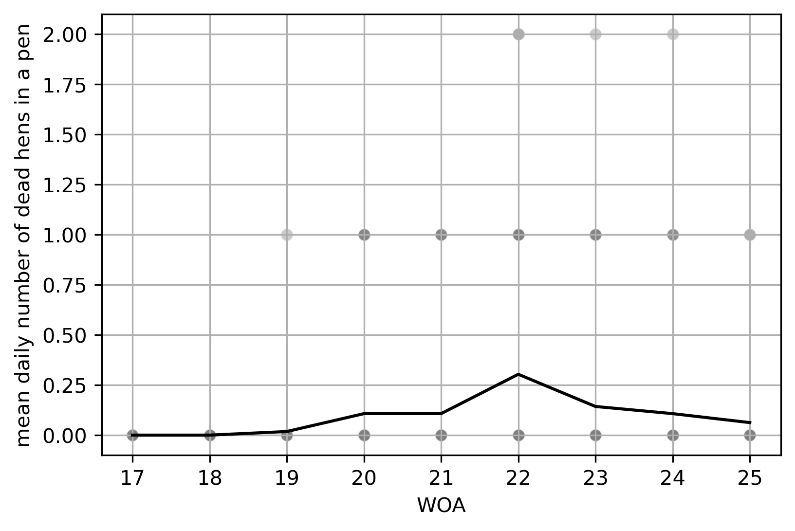


Figure S3 – The black line represents the weekly average of the daily number of dead hens across all pens and days, including a total of eight pens (each with 225 hens) and the first 54 days after the transfer to the laying barn (where WOA = week of age). Each point is a specific value for a pen on a specific day. The total number of early deaths was 46 (5 deaths/pen, except for three pens which had 6, 9 and 6 deaths respectively).


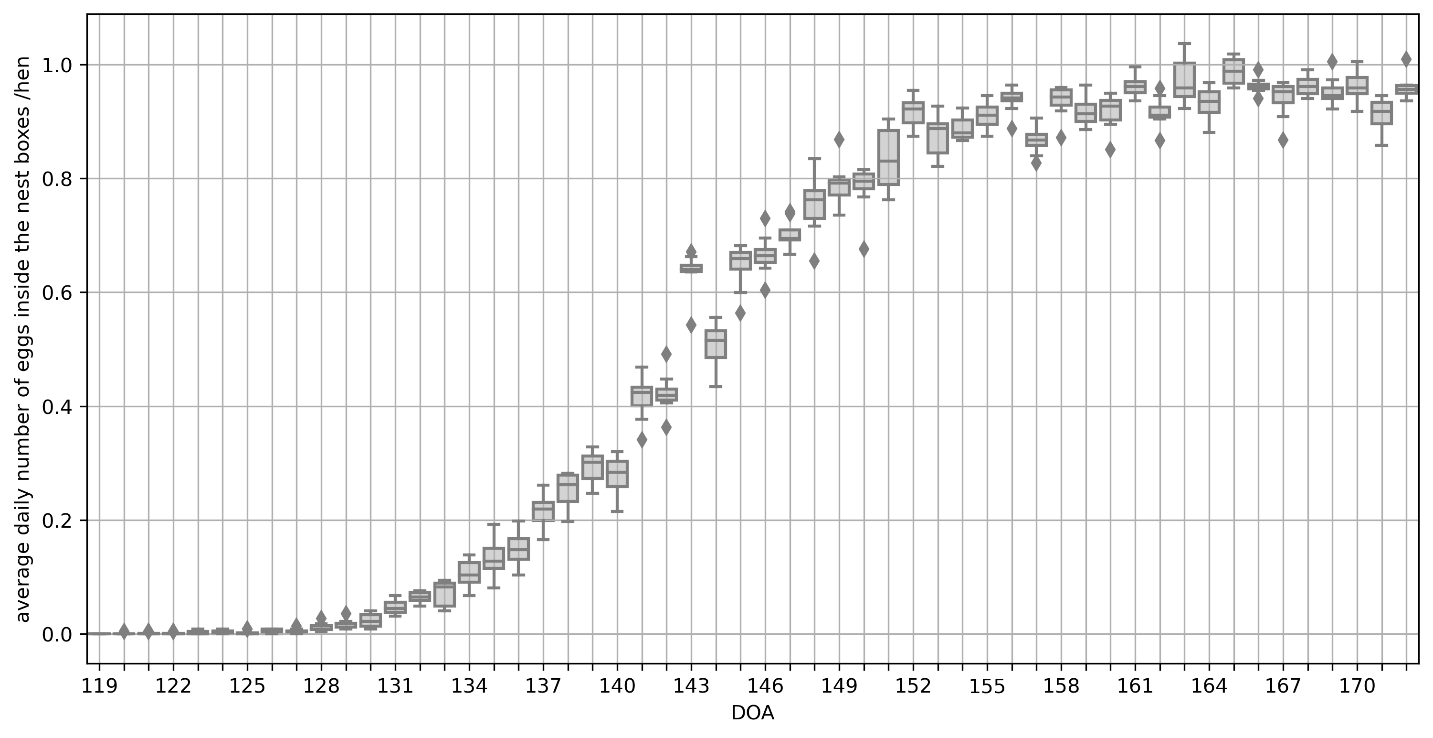

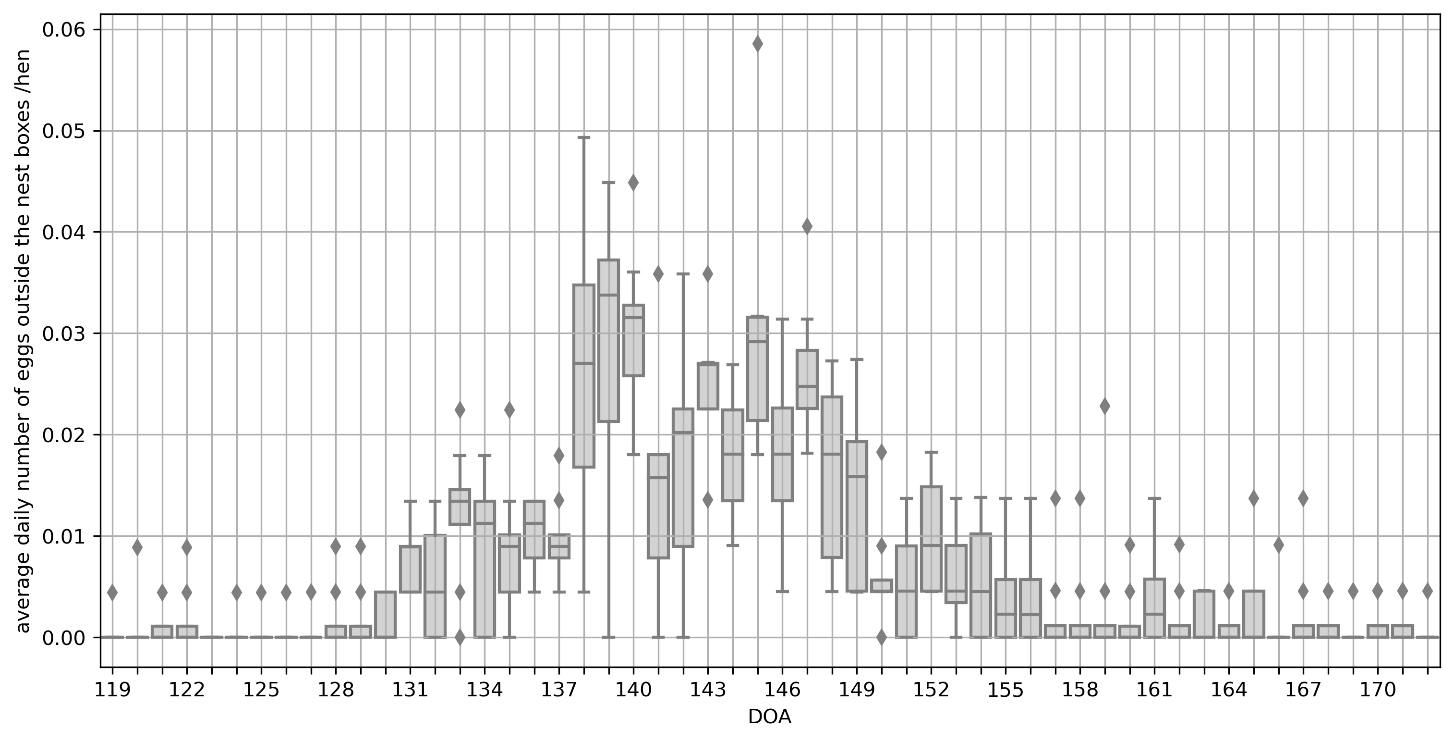


Figure S4 – Pen-level production data (for a total of eight pens), in terms of the average daily number of eggs laid (inside and outside the nest boxes) per hen over the first 54 days after the transfer to the laying barn.

**References**

1. Kaiser, H. F. The Application of Electronic Computers to Factor Analysis. *Educational and Psychological Measurement* **20**, 141–151 (1960).

2. Jollife, I. T. & Cadima, J. Principal component analysis: a review and recent developments. *Philosophical Transactions of the Royal Society A: Mathematical, Physical and Engineering Sciences* **374**, (2016).

3. Björklund, M. & Björklund, B. Be careful with your principal components. *Evolution (N Y)* **73**, 2151–2158 (2019).
